# Supplementary material for: Regulation of chitinase-3-like-1 in T cell elicits Th1 and cytotoxic responses to inhibit lung metastasis
Source: Nat Commun. 2018 Feb 5;9:503. doi: 10.1038/s41467-017-02731-6 (PMC5799380; doi:10.1038/s41467-017-02731-6)
Supplement: Supplementary file 1 — Supplementary Information [file 41467_2017_2731_MOESM1_ESM.pdf]

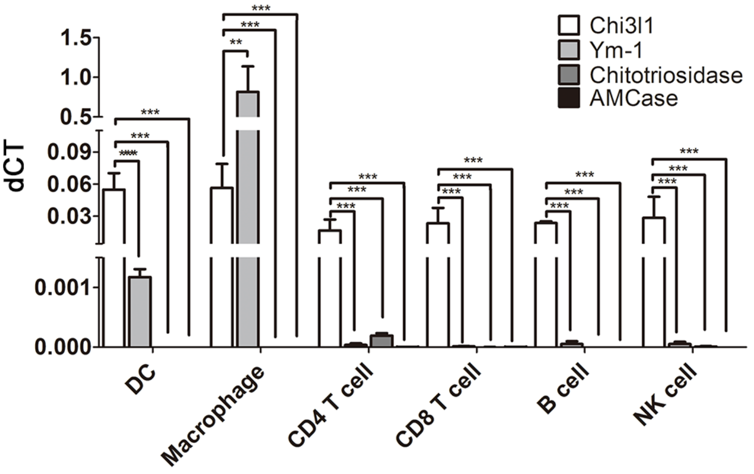

Supplementary Figure 1. mRNA expression of chitinase and chitinase-like protein in splenic immune cells. Each splenic immune cell population was sorted by FACS. Surface markers for sorting were $CD11c^{+}MHCII^{+}$: DC; $CD11c^{-}CD11b^{+}$: macrophage; $CD8^{+}CD4^{-}CD62L^{high}CD44^{low}$: naïve CD8 T cell; $CD4^{+}CD8^{-}CD62L^{high}CD44^{low}$: naïve CD4 T cell; $CD19^{+}$: B cell; and $CD3^{-}NK1.1^{+}$: NK cell. The mRNA levels of chitinase and chitinase like proteins were analyzed by RT-PCR. Each gene expression level was normalized to β-actin. Data are mean ± SD of four independent experiments. **p < 0.01, ***p < 0.001, ****p < 0.0001 (two-tailed Student's *t*-test).

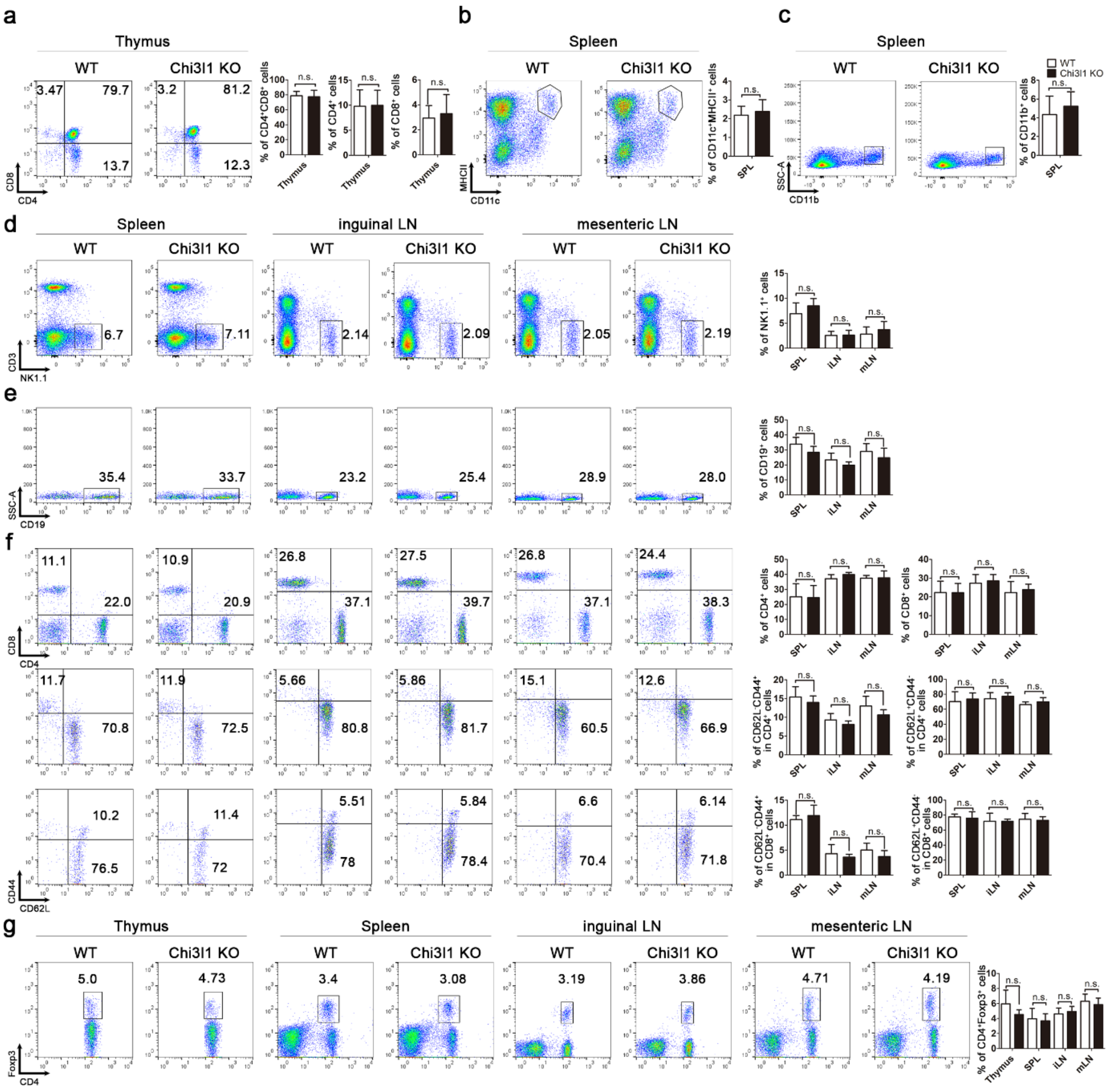

Supplementary Figure 2. Chi3l1 KO mice develops normal immune cell populations. The cells from indicated tissue of WT and Chi3l1 KO mice were isolated and stained with antibodies for flow cytometric analysis. (a) Percentages of CD4$^+$CD8$^+$ cells, CD4 single-positive cells, CD8 single-positive cells in the thymus were analyzed. (b-e) Percentages of dendritic cells (CD11c$^+$MHCII$^+$) and macrophages (CD11b$^+$), NK cells (NK1.1$^+$), and B cells (CD19$^+$) in the spleen was analyzed. (f) CD4 and CD8 T cells with activation marker of CD62L and CD44 in the spleen, inguinal lymph node, mesenteric lymph node was analyzed. (g) Foxp3 expressing regulatory T cells in the Thymus, spleen, inguinal lymph node, mesenteric lymph node was analyzed. Data are mean ± SD of 5 mice samples. n.s., not significant;

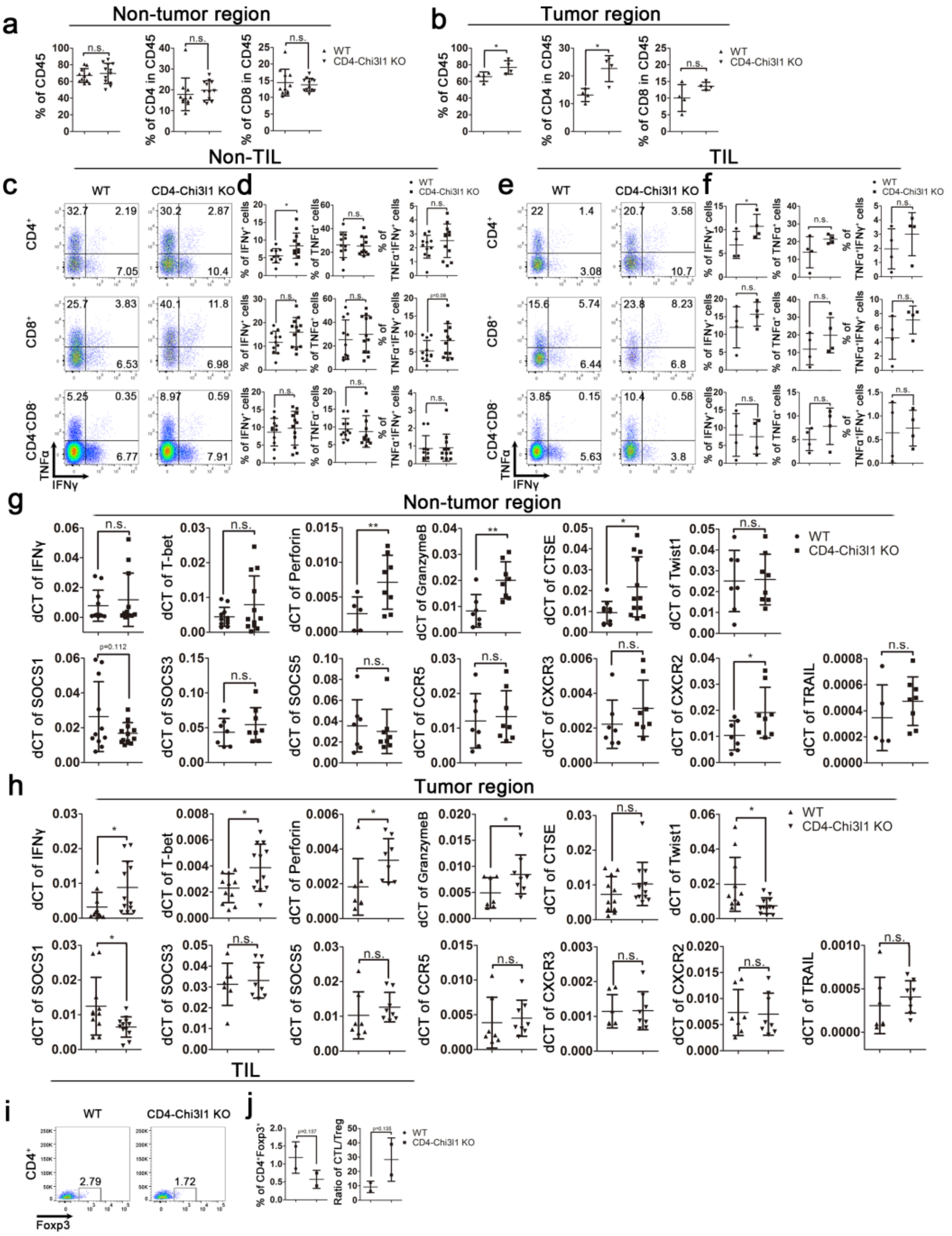

Supplementary Figure 3. Increase of tumor infiltrating effector T cells in the lung of CD4-Chi3l1 KO mice. The cells in the lung were isolated from non-tumor and tumor regions separately, then further analyzed by flow cytometry. (a, b) Percentage of $CD45^+$ cells, proportion of CD4 and CD8 in $CD45^+$ cells in non-tumor region (Non-TIL) and tumor region (TIL). (c-f) IFNγ and TNFα expressing $CD4^+$, $CD8^+$, $CD4^-CD8^-$ cells were analyzed in Non-TIL and TIL. (g, h) mRNA expression of genes related to cytotoxicity and Th1 effector functions was analyzed by quantitative RT-PCR. Each gene expression level was normalized to β-actin. Data are mean ± SD of two sets of independent experiments (n=8). (I) $CD4^+Foxp3^+$ cells in TIL. (j) % of $CD4^+Foxp3^+$ cells and ratio of CTL/Treg cell in TIL. Data are mean ± SD of two sets of independent experiments and each dot in graphs represent an individual mouse. n.s., not significant; *$p < 0.05$, **$p < 0.01$ (two-tailed Student's *t*-test).

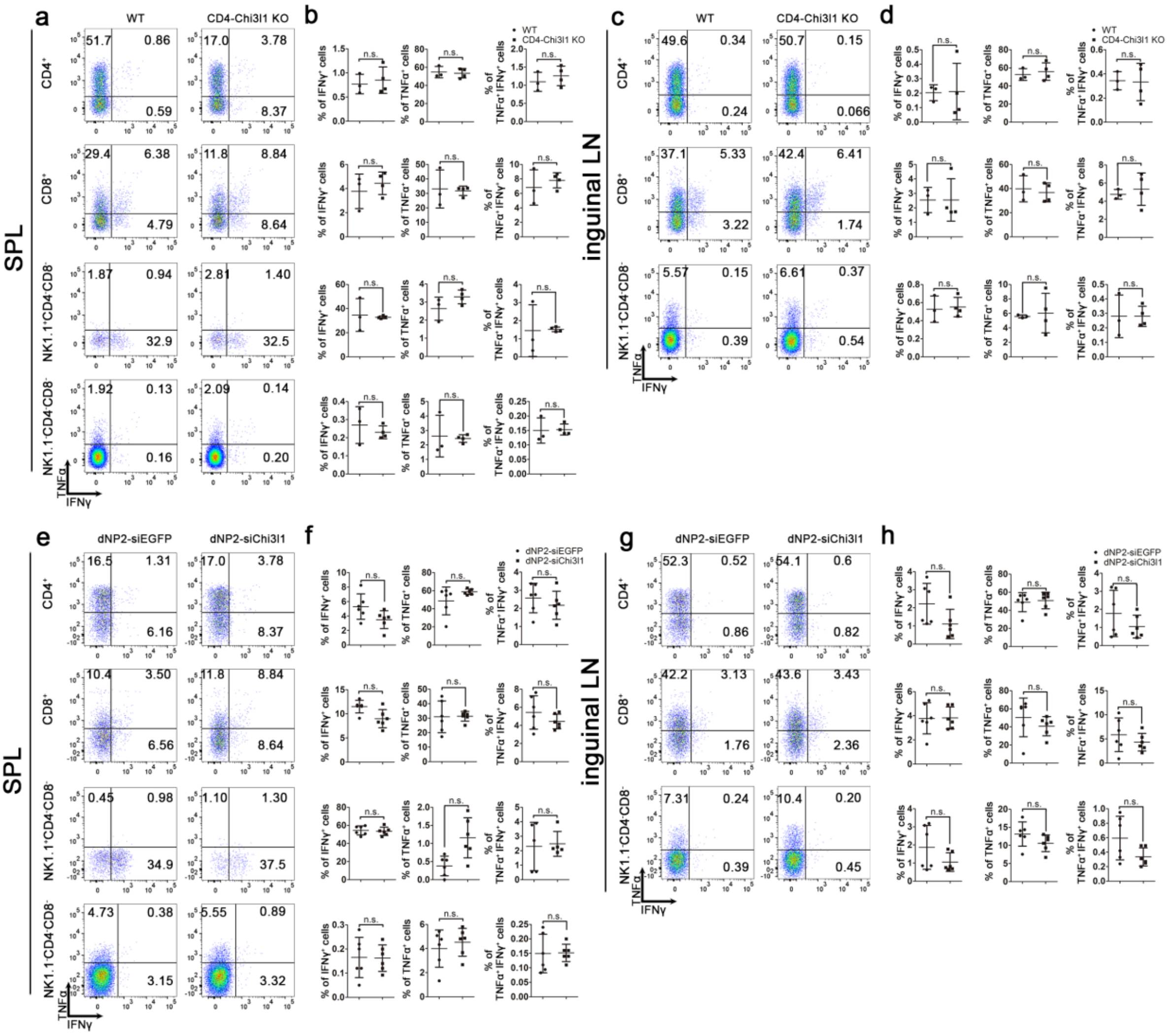

Supplementary Figure 4. Peripheral cytokine expressing immune cells in both CD4-Chi3l1 KO mice and dNP2-siChi3l1 treated mice. (a-d) IFNγ and TNFα expressing lymphocytes in the spleen and inguinal lymph node of WT and CD4-specific Chi3l1 KO mice following intravenous B16F10 melanoma injection were analyzed by flow cytometry. (e-h) IFNγ and TNFα expressing lymphocytes in the spleen and inguinal lymph node of dNP2-siEGFP or dNP2-siChi3l1 treated mice following intravenous B16F10 melanoma injection were analyzed. Data are mean ± SD of three sets of independent experiments and each dot in graphs represent an individual mouse. n.s., not significant;

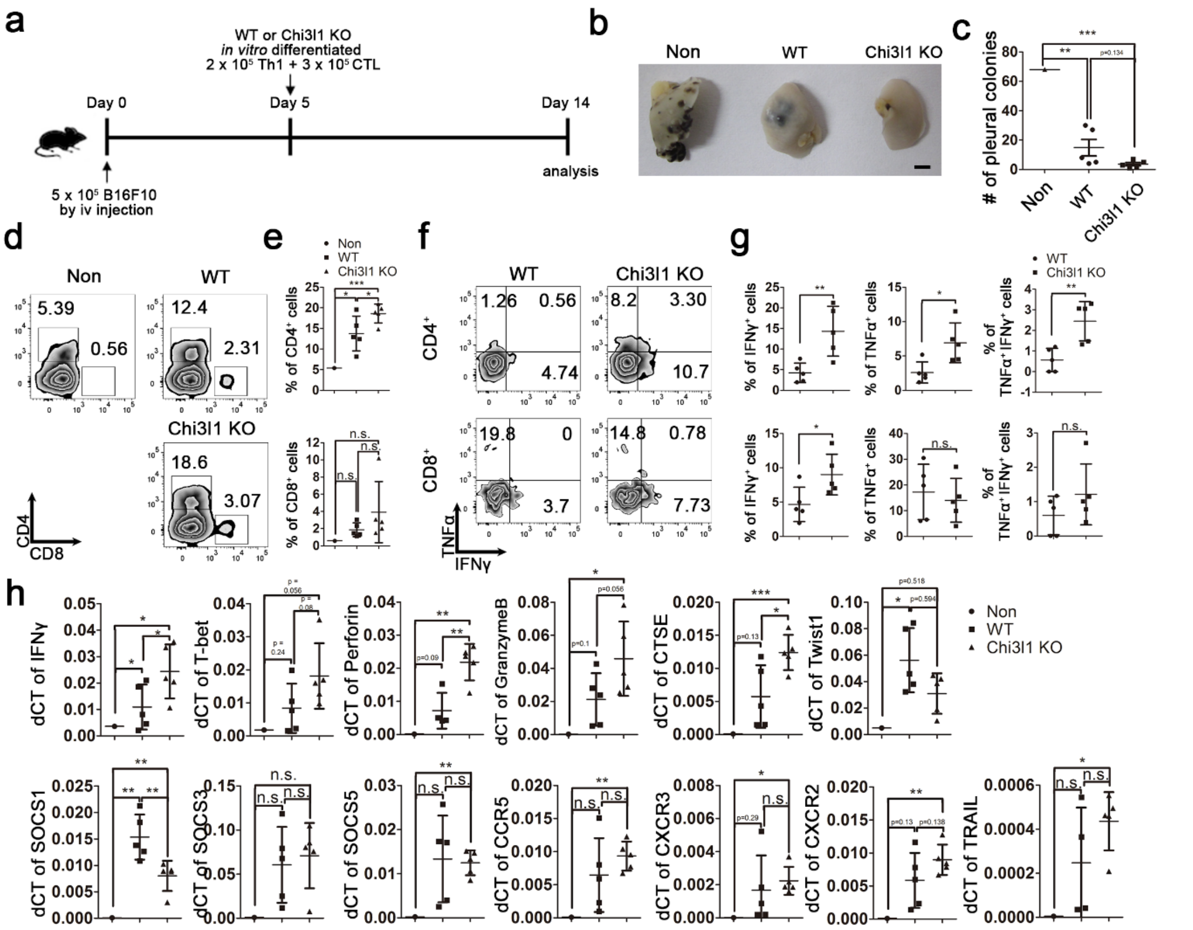

Supplementary Figure 5. Adoptive transfer of Chi3l1 KO Th1/CTL cells in RAG KO mice inhibits pulmonary metastasis. FACS-sorted WT and Chi3l1 KO naïve CD4 and CD8 T cells were differentiated into Th1 and CTL for 3 days. (a) RAG KO mice were injected with 5 x 10^5 B16F10 melanoma cells intravenously at day 0 with following transfer of WT or Chi3l1 KO Th1 (2 x 10^5) and CTL (3 x 10^5) at day 5. At day 14, the mice were sacrificed and analyzed. (b) Representative lung image. Scale bar, 2mm. (c) Number of pleural colonies on lungs. Data are mean ± SEM of 5 mice samples. (d, e) Proportion of CD4 and CD8 T cells in CD45 gating. (f, g) Flow cytometric analysis of IFNγ and TNFα expressing CD4 and CD8 T cells in the lung. (h) mRNA expression of genes related to cytotoxicity and Th1 effector functions was analyzed by quantitative RT-PCR. Each gene expression level was normalized to β-actin. Data are mean ± SD of 5 mice samples. n.s., not significant; *$p < 0.05$, **$p < 0.01$, ***$p < 0.001$ (two-tailed Student's *t*-test).

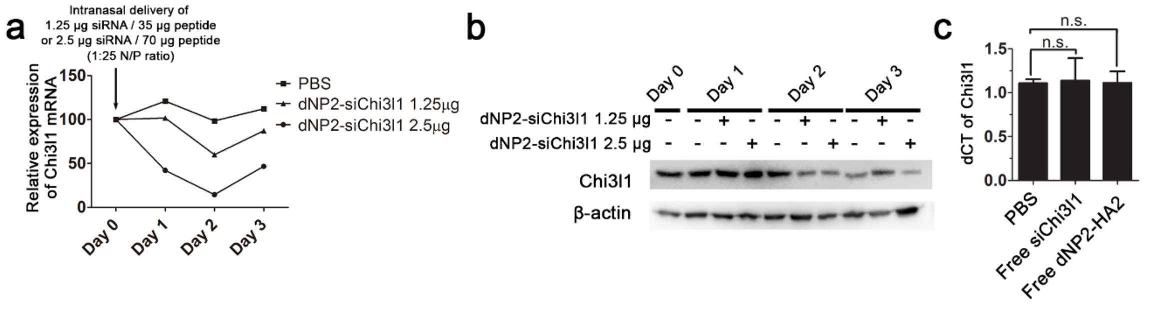

Supplementary Figure 6. Targeted knockdown of Chi3l1 by dNP2-siChi3l1 complex in dose dependent manner. (a) Chi3l1 mRNA level in the lung was analyzed by RT-PCR. Chi3l1 mRNA expression level was normalized to β-actin, and represented as relative expression of Chi3l1 mRNA to day 0. (b) Chi3l1 protein level in the lung was analyzed by Western blotting. Data are from one time experiment. (c) Chi3l1 mRNA expression in the lung was analyzed on day 2 following free siRNA or free peptide treatment. Chi3l1 mRNA expression level was normalized to β-actin. Data are mean ± SD of 3 mice samples. n.s., not significant;

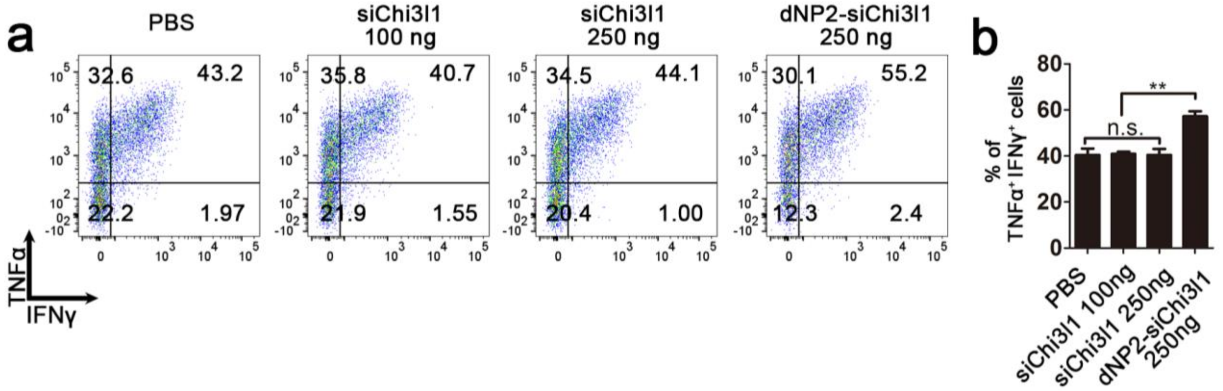

Supplementary Figure 7. dNP2 is required for Chi3l1 knockdown by siChi3l1 in T cells. MACS-sorted WT naive CD4 T cells differentiated into Th1 cells with the indicated concentrations of siChi3l1 or dNP2-siChi3l1 complex. (a) Intracellular level of IFNγ and TNFα expression level was analyzed by flow cytometry. (b) % of $TNF\alpha^{+}IFN\gamma^{+}$ cells were represented as bar graph. Data are mean ± SD of 3 mice samples. n.s., not significant; **$p < 0.01$ (two-tailed Student's *t*-test).

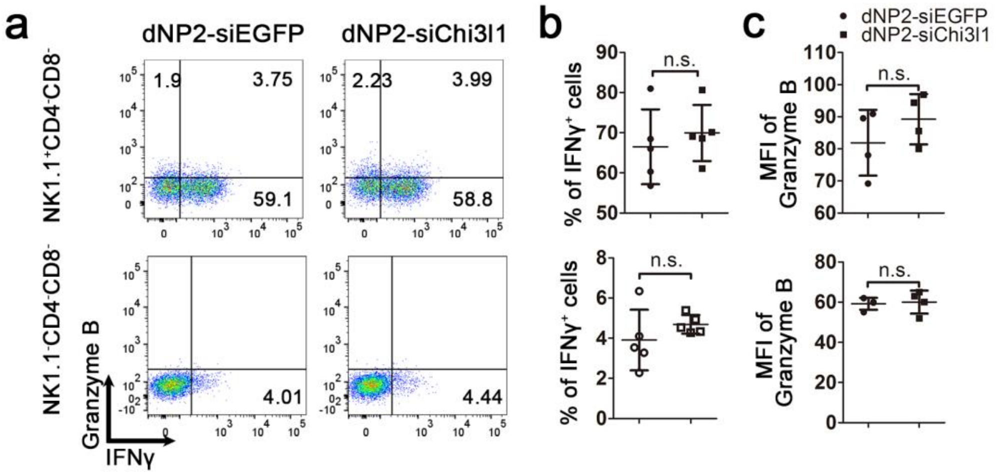

Supplementary Figure 8. NK cells and non-lymphocytic cells were not affected by dNP2-siChi3l1 treatment in mice. (a) IFNγ and Granzyme B expression level was analyzed by flow cytometry in NK cells (NK1.1^{+}CD4^{-}CD8^{-}) and the non-lymphocytic population (NK1.1^{-}CD4^{-}CD8^{-}) from dNP2-siEGFP or dNP2-siChi3l1-treated mice. (b, c) Percentage of IFNγ and mean fluorescence intensity (MFI) of Granzyme B expressing cells were analyzed. Data are mean ± SD of 5 mice samples. n.s., not significant;

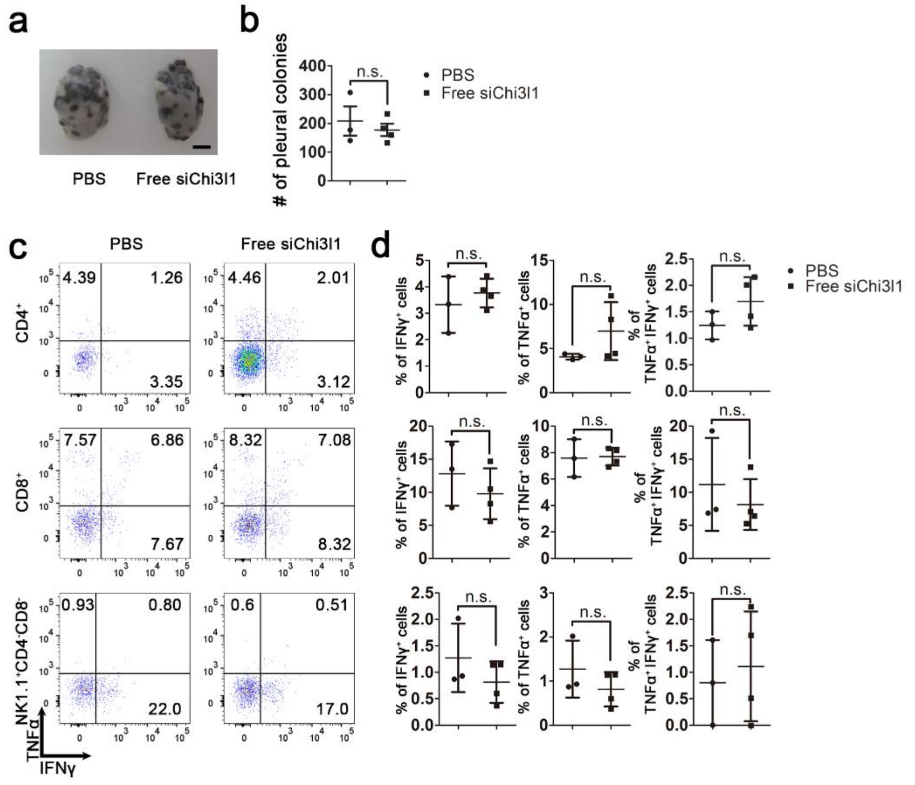

Supplementary Figure 9. Free siChi3l1 itself treatment does not inhibit pulmonary metastasis. (a) Representative lung image from intranasal PBS or free siChi3l1-treated mice with intravenous B16F10 melanoma injection. Scale bar, 2mm. (b) Number of pleural colonies in the lung of PBS or free siChi3l1-treated mice. Data are mean ± SEM and each dot in graphs represent an individual mouse. (c) Flow cytometric analysis of IFNγ and/or TNFα expressing CD4 and CD8 T cells, and NK cells. (d) Scattered graph of representative results. Data are mean ± SD and each dot in graphs represent an individual mouse. n.s., not significant;

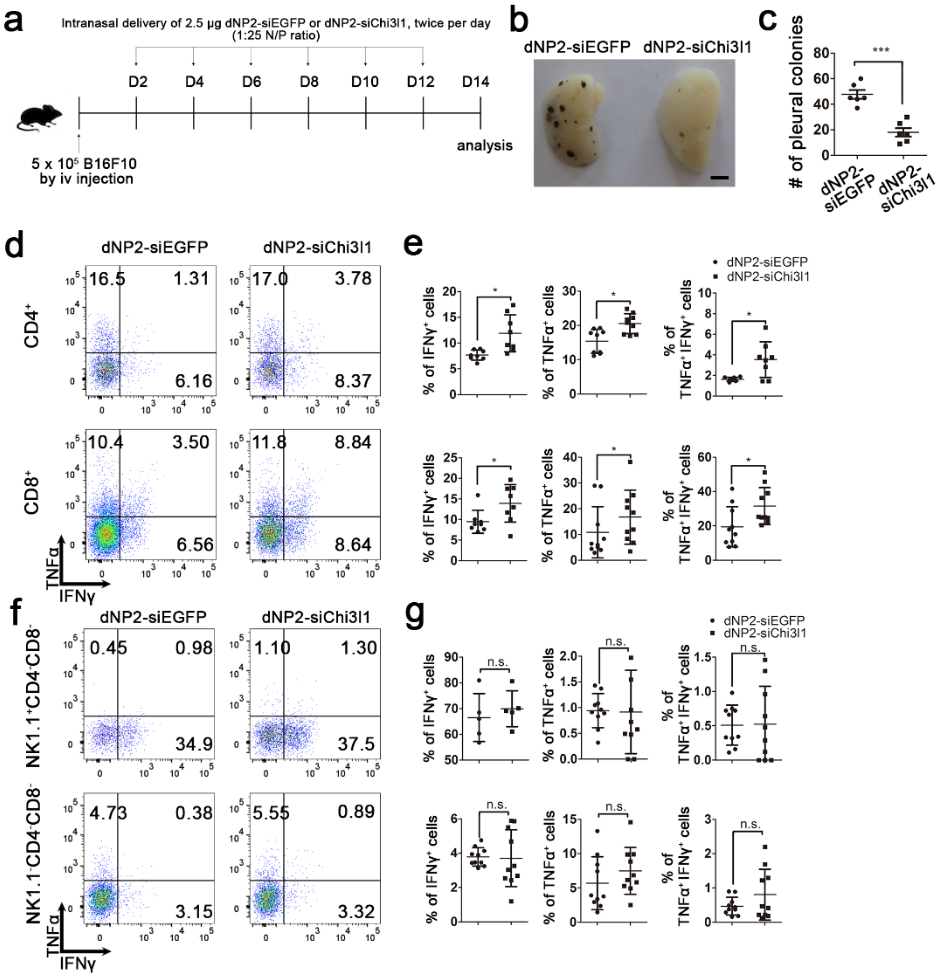

Supplementary Figure 10. Therapeutic effect of dNP2-siChi3l1 complex treatment inhibits pulmonary metastasis. (a) Experimental therapeutic treatment scheme of dNP2-siChi3l1 complex in pulmonary melanoma metastasis model. (b) Representative lung image from dNP2-siEGFP- or dNP2-siChi3l1-treated mice with intravenous B16F10 melanoma injection. Scale bar, 2mm. (c) Number of pleural colonies in the lung. Data are mean ± SEM of three sets of independent experiments and each dot in graphs represent an individual mouse. (d-g) Flow cytometric analysis of IFNγ and TNFα expressing CD4 and CD8 T cells or NK cells and non-lymphocytic cells (NK1.1$^-$CD4$^-$CD8$^-$). Data are mean ± SD of three sets of independent experiments and each dot in graphs represent an individual mouse. n.s., not significant; *p < 0.05, ***p < 0.001 (two-tailed Student's *t*-test).

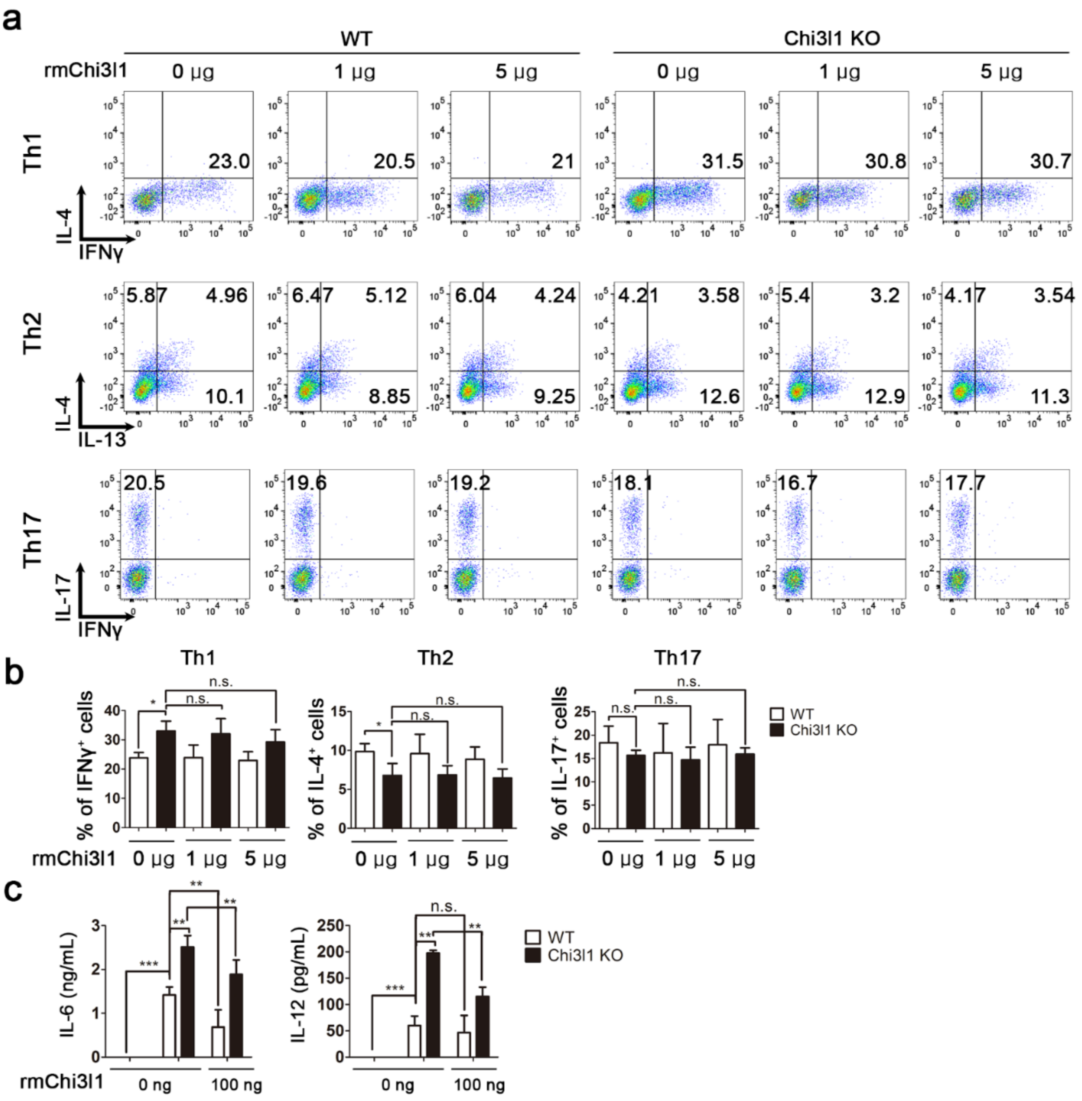

Supplementary Figure 11. Exogenous Chi3l1 protein treatment does not effect on Th1 differentiation. MACS-sorted WT and Chi3l1 KO naïve CD4 T cells were differentiated into Th1, Th2, and Th17 cells under lineage-specific cytokine conditions with the indicated concentration of recombinant mouse Chi3l1 (rmChi3l1) protein. (a, b) Lineage-specific cytokine expression was analyzed by flow cytometry. (c) Peritoneal macrophages from WT and Chi3l1 KO mice were isolated and stimulated with LPS for 12 hours in vitro. IL-6 and IL-12 level in culture supernatant was analyzed by ELISA. Data are mean ± SD of three sets of independent experiments. n.s., not significant; *p < 0.05, **p < 0.01, ***p < 0.001 (two-tailed Student's *t*-test).

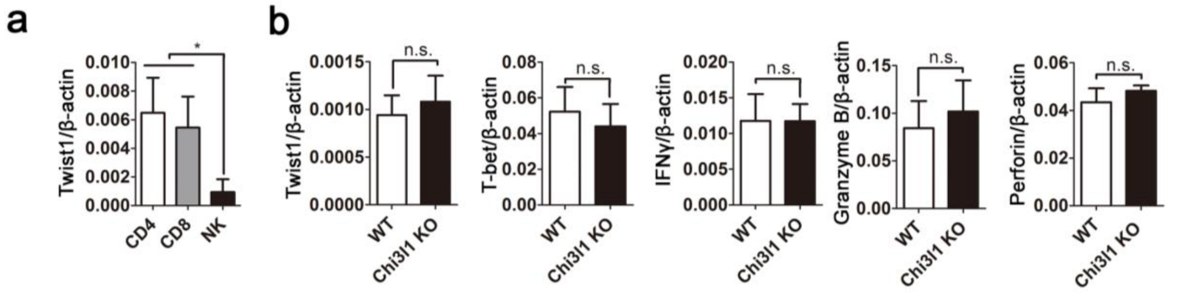

Supplementary Figure 12. Th1 or CTL function-related gene expressions in NK cells. (a) Twist1 mRNA expression in FACS-sorted naïve CD4, CD8, and NK cells. (b) mRNA levels of Twist1, T-bet, IFNγ, Perforin, and Granzyme B in activated WT and Chi3l1 KO NK cells. Each gene expression level was normalized to β-actin. Data are mean ± SD of three sets of independent experiments. n.s., not significant; *p < 0.05 (two-tailed Student's *t*-test).

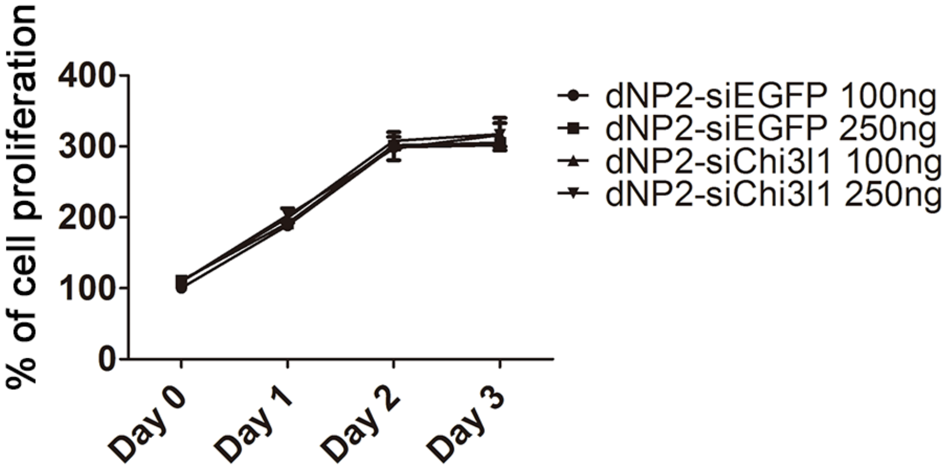

Supplementary Figure 13. dNP2-siChi3l1 complex does not directly inhibit B16F10 melanoma cell growth. 4 x 10^4 B16F10 melanoma cells were seeded into 96-well plates with 100 or 250 ng dNP2-siEGFP or dNP2-siChi3l1. Based on CCK-8 staining result, % of cell proliferation was calculated as increased live cell percentage relative to the value at day 0. Data are mean ± SD of three sets of independent experiments.

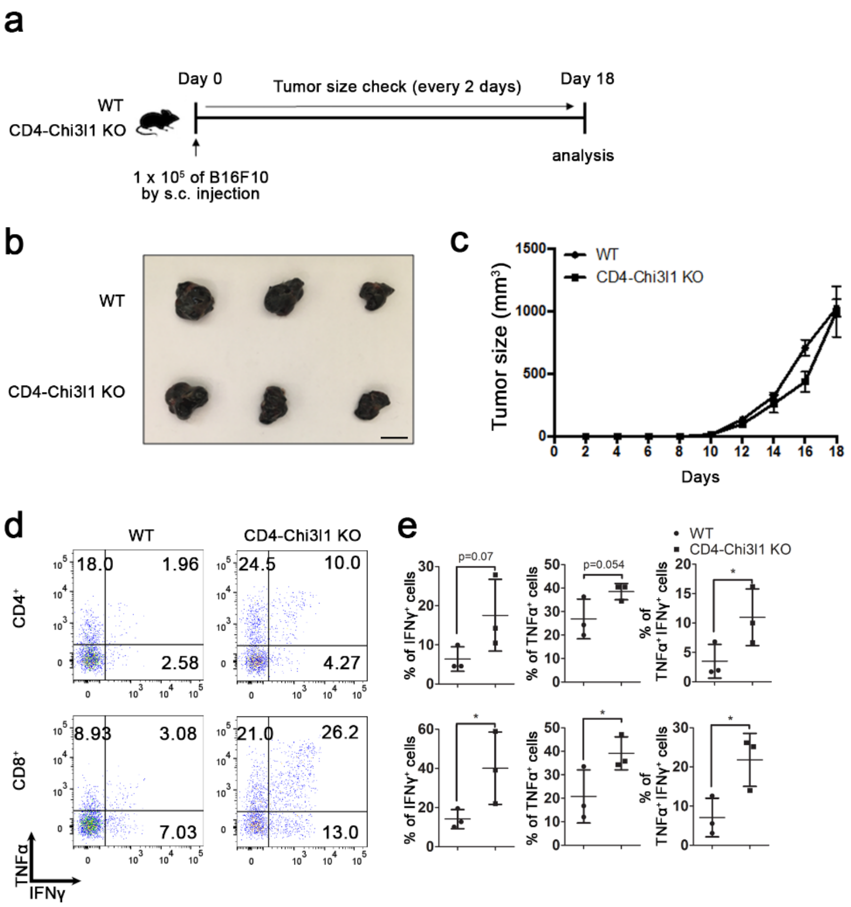

Supplementary Figure 14. Increased tumor infiltrating IFNγ-producing T cells in subcutaneous solid tumor of CD4-Chi3l1 KO mice. (a) WT and Chi3l1 KO mice were implanted with 1 x 10^5 B16F10 melanoma cells by subcutaneous injection. Tumor size was measured every 48 hours with a caliper until Day 18. (b) Representative image of isolated tumor tissues from WT and CD4-Chi3l1 KO mice on day 18. Scale bar, 10mm. (c) Tumor size measurement with a caliper. Data are mean ± SEM of three mice samples. (d, e) Flow cytometric analysis of tumor infiltrating IFNγ and/or TNFα producing CD4 and CD8 T cells. Data are mean ± SD of 3 mice samples. *p < 0.05 (two-tailed Student's *t*-test).

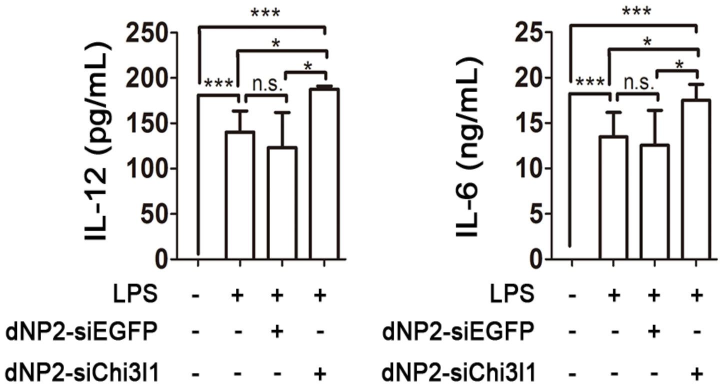

Supplementary Figure 15. dNP2-siChi3l1 treatment in macrophages enhances IL-12 and IL-6. Mouse peritoneal macrophages were isolated and stimulated with LPS for 12 hours with or without 250 ng of dNP2-siEGFP or dNP2-siChi3l1 treatment. IL-12 and IL-6 production in culture supernatant was measured by ELISA. Data are mean ± SD of at three sets of independent experiments. n.s., not significant; *p < 0.05, ***p < 0.001 (two-tailed Student's *t*-test).

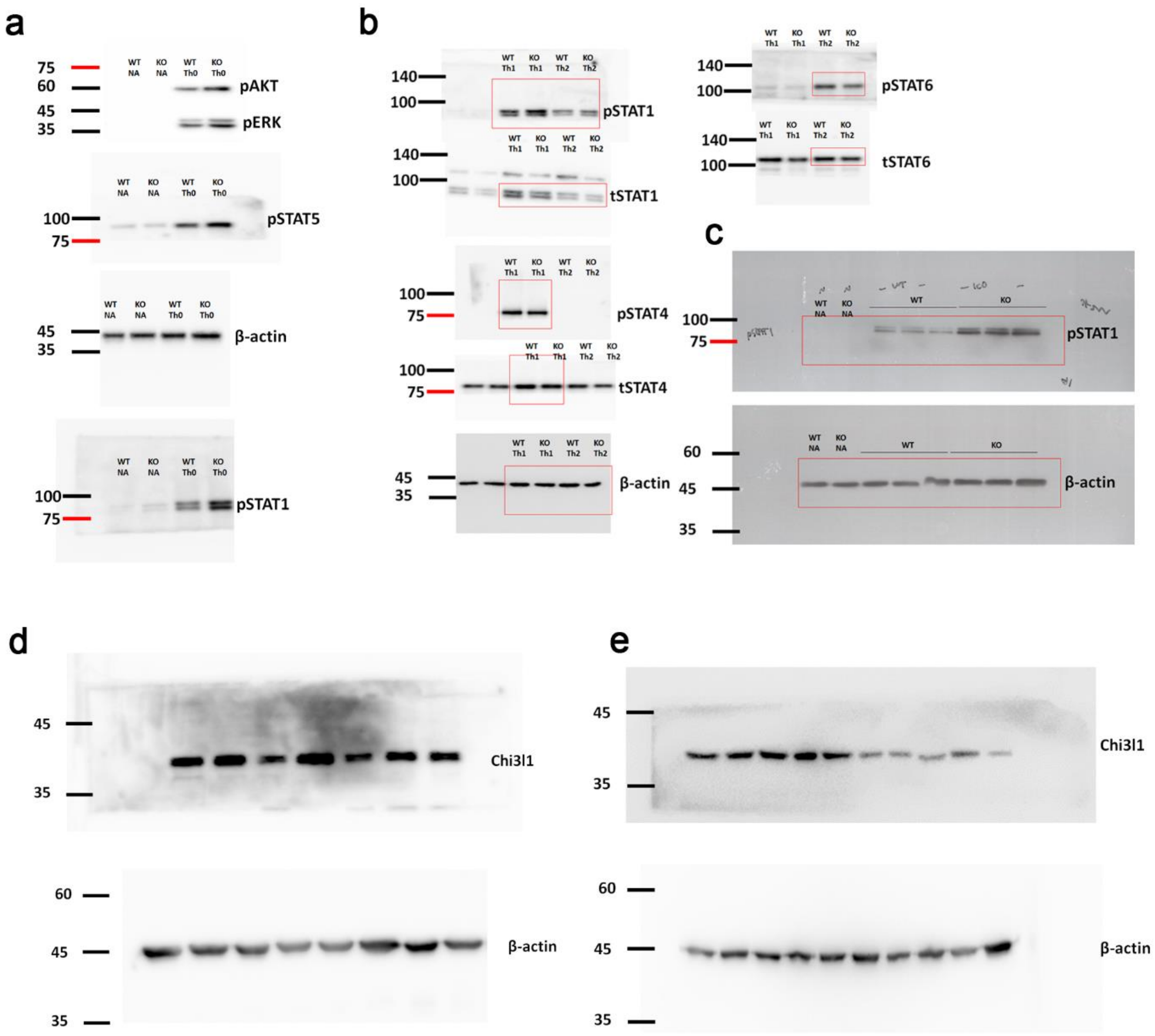

Supplementary Figure 16. Raw western blotting images used in figures. (a-e) The full western blot images of Figure 2g (a), Figure 3 e (b), Figure 3h (c), Figure 7g (d), and Supplementary Fig. 7 (e).

	**Forward 5' to 3'**	**Reverse 3' to 5'**
Actb	TGTCCCTGTATGCCTCTGGT	CACGCACGATTTCCCTCTC
Chi3l1	GTACAAGCTGGTCTGCTACTTC	ATGTGCTAAGCATGTTGTCGC
Tbx21	AGCAAGGACGGCGAATGTT	GGGTGGACATATAAGCGGTTC
Runx3	GACTCCTTCCCCAACTATACACC	GTGCTCGGGTCTCGTATGAA
Ifng	ATGAACGCTACACACTGCATC	CCATCCTTTTGCCAGTTCCTC
Gata3	GAAGGCATCCAGACCCGAAAC	ACCCATGGCGGTGACCATGC
Il4	GGTCTCAACCCCCAGCTAGT	GCCGATGATCTCTCTCAAGTGAT
Il13	CAGCCTCCCCGATACCAAAAT	GCGAAACAGTTGCTTTGTGTAG
Il5	CTCTGTTGACAAGCAATGAGACG	TCTTCAGTATGTCTAGCCCCTG
Il10	GCTCTTACTGACTGGCATGAG	CGCAGCTCTAGGAGCATGTG
Junb	TCACGACGACTCTTACGCAG	CCTTGAGACCCCGATAGGGA
Rorgt	GACCCACACCTCACAAATTGA	AGTAGGCCACATTACACTGCT
Il17	TTTAACTCCCTTGGCGCAAAA	CTTTCCCTCCGCATTGACAC
Il21	GGACCCTTGTCTGTCTGGTAG	TGTGGAGCTGATAGAAGTTCAGG
Gmcsf	GGCCTTGGAAGCATGTAGAGG	GGAGAACTCGTTAGAGACGACTT
Batf	GTTCTGTTTCTCCAGGTCC	GAAGAATCGCATCGCTGC
Socs1	CTGCGGCTTCTATTGGGGAC	AAAAGGCAGTCGAAGGTCTCG
Socs3	ATGGTCACCCACAGCAAGTTT	TCCAGTAGAATCCGCTCTCCT
Socs5	GAGGGAGGAAGCCGTAATGAG	CGGCACAGTTTTGGTTCCG
Perforin	GAGAAGACCTATCAGGACCA	AGCCTGTGGTAAGCATG
Gzmb	CCTCCTGCTACTGCTGAC	GTCAGCACAAAGTCCTCTC
Il13ra2	ACCGAAATGTTGATAGCGACAG	ACAATGCTCTGACAAATGCGTA
Chit1	TGGGCAGGTGTGATGACTCT	CCCTGGGAAAGAACCGAACTG
Amcase	CTGCGTCAGTATGGGTTTGAT	TGGGCCTGTTGCTCTCAATAG
Ym-1	ACCTGCCCCGTTCAGTGCCAT	CCTTGGAATGTCTTTCTCCACAG
Foxp3	CCCATCCCCAGGAGTCTTG	ACCATGACTAGGGGCACTGTA
Eomes	TCATCGCTGTGACGGCCTACCA	GGGGAATCCGTGGGAGATGGAGT
Tnfsf10	ATGGTGATTTGCATAGTGCTCC	GCAAGCAGGGTCTGTTCAAGA
Ccr5	TTTTCAAGGGTCAGTTCCGAC	GGAAGACCATCATGTTACCCAC
Cxcr3	TACCTTGAGGTTAGTGAACGTCA	CGCTCTCGTTTTCCCCATAATC
Cxcr2	ATGCCCTCTATTCTGCCAGAT	GTGCTCCGGTTGTATAAGATGAC
Ctse	GACATCAGTCCCTTCGGAAGA	AGGGGTTCATTGACACTCGAATA
Twist1	GGACAAGCTGAGCAAGATTCA	CGGAGAAGGCGTAGCTGAG

Supplementary Table 1. List of primers
